# Thiocyanate-Treated Perovskite-Nanocrystal-Based Light-Emitting Diodes with Insight in Efficiency Roll-Off

**DOI:** 10.3390/ma13020367

**Published:** 2020-01-13

**Authors:** Fang Chen, Karunakara Moorthy Boopathi, Muhammad Imran, Simone Lauciello, Marco Salerno

**Affiliations:** 1 Optoelectronics, Istituto Italiano di Tecnologia, via Morego 30, 16163 Genova, Italy; fang.chen@iit.it; 2 Dipartimento di Chimica e Chimica Industriale, Università degli Studi di Genova, via Dodecaneso 31, 16146 Genova, Italy; 3 Nanochemistry Department, Istituto Italiano di Tecnologia, via Morego 30, 16163 Genova, Italy; karunakara.boopathi@iit.it (K.M.B.); muhammad.imran@iit.it (M.I.); simone.lauciello@iit.it (S.L.); 4 Materials Characterization Facility, Istituto Italiano di Tecnologia, via Morego 30, 16163 Genova, Italy

**Keywords:** perovskite nanocrystal, light emitting diodes, thiocyanate, efficiency roll-off, conductive atomic force microscopy

## Abstract

Light emitting diodes (LED) based on halide perovskite nanocrystals (NC) have received widespread attention in recent years. In particular, LEDs based on CsPbBr_3_ NCs were the object of special interest. Here, we report for the first time green LED based on CsPbBr_3_ NCs treated with ammonium thiocyanate solution before purification with polar solvent. The champion device fabricated based on the treated CsPbBr_3_ NCs showed high efficiency and high stability during operation as well as during storage. A study on morphology and current distribution of NC films under applied voltages was carried out by conductive atomic force microscopy, giving a hint on efficiency roll-off. The current work provides a facile way to treat sensitive perovskite NCs and to fabricate perovskite NC-based LED with high stability. Moreover, the results shed new light on the relation between film morphology and device performance and on the possible mechanism of efficiency roll-off in NC LED.

## 1. Introduction

All-inorganic halide perovskite has attracted much attention since successful synthesis of CsPbX_3_ (X = Cl, Br, I) NC in 2015 [1]. Owing to their excellent photoluminescence (PL) performance [2] and facile synthesis in solution [3], CsPbX_3_ NCs, especially CsPbBr_3_, are currently the subject of wide exploration in LED [4]. In comparison with their contemporary organic-inorganic hybrid halide perovskites, CsPbX_3_ NCs exhibit higher stability and narrower emission width [5]. Currently, the best halide perovskite nanocrystal-based LED with green emission has achieved a maximum external quantum efficiency (EQE) of 16.48%, based on NCs containing mixed cations of cesium and formamidine [6]. However, progress made in all-inorganic halide-perovskite nanocrystal-based LED relatively lags behind.

Various synthesis strategies for CsPbBr_3_ NCs have been extensively exploited based on different source materials, ligands and reaction conditions. Continuous progresses are being made in applying CsPbBr_3_ NCs synthesized by different methods into LED fabrication. Firstly, based on the first synthesis route of CsPbBr_3_ NCs [1], CsPbBr_3_ NCs synthesized by PbBr_2_ and Cs_2_CO_3_ have obtained a fast development in EQE of corresponding LEDs. For example, EQEs of 0.26% [7] and 5.7% [8] have been achieved by optimizing transporting layers based on the initial EQE of 0.026% [7]. By optimizing ligand washing process, the EQE was increased to 1.7% [9]. Via ligand exchange in post-synthesis, EQE of 3.0% [10] and 4.33% [11] were realized, respectively, with didodecyldimethyl ammonium bromide (DDAB) and conjugation molecular. By combination of ligand exchange with DDAB and optimized ligand washing strategy, the EQE was further increased up to more than 8.0% [12]. However, it is tedious to perform ligand exchange and the productivity of NCs after ligand exchange is rather low. Application of the CsPbBr_3_ NCs into LEDs with inverted structure has developed from initial EQE of 0.19% [13] up to 4.63% [14] by optimizing band alignment of transporting layers and introducing localized surface plasma. Secondly, when the source material of cesium is replaced with cesium stearate, performance of corresponding LEDs has also received a steady enhancement. For instance, starting from initial EQE of 0.12% [15], further works have increased the EQE to 2.21% by forming composite with CsPb_2_Br_5_ [16], and 6.27% by rational washing procedure of the CsPbBr_3_ NCs [17]. Trial of the CsPbBr_3_ NCs with DDAB ligand exchange in inverted LED structure has obtained a maximum EQE of 0.58% [18]. In addition, cesium acetate was also applied into synthesis of CsPbBr_3_ NCs. Together with octylphosphonic acid as ligand, EQE of 6.5% [19] was achieved. Thirdly, some work involved the use of new types of halide sources in synthesis of the CsPbBr_3_ NCs, e.g., tetraoctylammonium bromide (TOABr), NH_4_Br, trioctylphosphine-Br_2_ and benzoyl bromide. For instance, LED based on CsPbBr_3_ NCs prepared with TOABr as halide source and oleic acid as ligand obtained a maximum EQE of 0.325% [20]. Through combining mixed cation, ligand exchange, rational washing and surface passivation in treatment of CsPbBr_3_ NCs, LED based on the CsPbBr_3_ NCs, synthesized with PbBr_2_, Cs_2_CO_3_ and TOABr, has obtained a maximum EQE of 16.48% [6]. In another case, LEDs based on CBP NCs using NH4Br [21] and trioctylphosphine-Br_2_ [22] in synthesis have also been reported, with maximum EQEs at 1.2% and 2.5%, respectively. However, despite that CBP NCs prepared with benzoyl bromide as halide source have achieved a high PL quantum yield (PLQY) of 92% in solution [23], there has been no report on corresponding LED.

Passivation of defects in either perovskite thin film or perovskite NCs play an important role. For example, with methylammonium bromide additive passivating non-radiative defect sites in CsPbBr_3_ thin film, a milestone EQE of 20.3% was realized [24]. For another example, with inorganic halide ionic compounds passivating CsPbBr_3_ NCs before purification with anti-solvent, the up-to-date highest EQE for perovskite NCs has been achieved [6]. Salts containing thiocyanate (SCN) have been demonstrated to be able to increase grain size and reduce defects in perovskite thin film [25] and have shown beneficial effects on stability [26], device performance and suppression of hysteresis in solar cell [27]. SCN, as a kind of pseudohalide, has also presented efficient passivation effect on perovskite NCs [28]. For instance, Alivisatos and coworkers reported that SCN improves PLQY and stability of CBP NCs by surface passivation [29], and proposed a principle to passivate CsPbBr_3_ NCs with softer, anionic and X-type Lewis bases [30]. Recently, Lu et al. [31] reported red-emitting LEDs based on CsPbI_3_ NCs treated with ammonium SCN. However, there has been no report on green-emitting LED based on SCN-treated CsPbBr_3_ NCs.

Efficiency roll-off at high current density in perovskite LED has started to receive attention. Auger recombination has been determined as a reason for this effect [32], and some solutions have been proposed correspondingly. For instance, core-shell structure was shown to be beneficial to increase threshold current of efficiency roll-off in QD LED [33]. In another work, in 2D perovskite with funnel structure, the roll-off was demonstrated to be reduced through increasing content ratio of low-bandgap perovskite [32], which concentrates the injected electrons/holes and plays the major role in light emission. For 3D perovskite NCs, material degradation [34] and destruction of film morphology [35] at high current density were predicted to play critical roles in the roll-off. However, there has been no direct proof reported for this hypothesis.

In this work, green LEDs based on CsPbBr_3_ treated with ammonium SCN solution before purification with polar solvent were fabricated for the first time. The LEDs show high efficiency compared with LEDs based on CsPbBr_3_ NCs without DDAB ligand exchange, which is tedious and of low productivity in NC preparation [36]. The champion device also shows high stability during operation and during storage. Investigations on morphology and current distribution of NC films with structure similar to the final device, under applied voltages, were performed by conductive atomic force microscopy (AFM), to illustrate the possible mechanisms of better performance in the champion device. With this work, we provide a facile treatment for achieving stable perovskite NC LED and present some insight in the possible mechanism of efficiency roll-off in NC LED.

## 2. Materials and Methods

### 2.1. Materials

Ethyl acetate, ammonium thiocyanate and 1-Butanol were purchased from Sigma-Aldrich (Milan, Italy) and were stored in glovebox. NC solutions synthesized according to the previous report [23] were treated in such a way that 300 µL as-prepared CsPbBr_3_ NC solution was firstly added into a vial. Secondly, an amount of ammonium thiocyanate (SCN) solution, for example, 10, 30 or 50 µL, was dropped into the vial. Thirdly, 900 µL EA was gradually added into the solution, and the obtained solution was left under hood for 5 min. Then, the treated CsPbBr_3_ NC solution was centrifuged at 6000 rpm for 10 min. The supernatant was discarded, and the precipitate was re-dispersed in 300 µL hexane for film and device fabrication. Here, SCN solution was prepared by dissolving 10 mg ammonium thiocyanate in a mixture of 4 mL 1-butanol and 6 mL toluene.

### 2.2. Device Preparation

LEDs were fabricated on patterned substrates of glass coated with ~200 nm thick indium tin oxide (ITO). The substrates were cleaned in an ultrasonic bath using detergent, deionized water, isopropanol and deionized water, sequentially. Prior to depositing hole transporting layers, the ITO glass substrates were further treated with oxygen plasma for 300 s at 30 W. A layer of poly(ethylene dioxythiophene): polystyrenesulphonate (PEDOT:PSS) was spin-coated onto the cleaned ITO glass substrates at 4000 rpm and annealed at 150 °C for 30 min in hood. Poly[*N*,*N*′-bis(4-butylphenyl)-*N*,*N*′-bisphenylbenzidine] (poly-TPD) was spin-coated onto the PEDOT:PSS layer at 5000 rpm and annealed at 120 °C for 20 min in glovebox (GB). The total thickness of PEDOT:PSS and poly-TPD was ~60 nm. Here, the poly-TPD solution was prepared in chlorobenzene at 8 g/L in GB. As the film cool down, CsPbBr_3_ NC solutions were spin-casted at 2000 rpm and annealed at 70 °C for 15 min. The thickness was ~30 nm. Then, electron transporting layer 1,3,5-Tris(1-phenyl-1H-benzimidazol-2-yl)benzene (TPBi) (~45 nm) and electrode LiF (~1.5 nm) and Al (~100 nm) were thermal evaporated at rate of 1.5, 0.2, 2 nm/s, respectively. Finally, the devices were encapsulated with cover glasses and encapsulation oil. The devices were treated by UV light for 15 min to solidify the encapsulation oil in GB.

### 2.3. Characterization

The NC solutions were characterized by a transmission electron microscope (TEM) JEOL-1100 (Jeol, Tokyo, Japan) at 100 kV and low resolution (after drop-casting onto carbon-coated copper grids) and by X-ray diffraction (XRD) on a Empyrean X-ray diffractometer (PANanalytical, Almelo, the Netherlands) equipped with a 1.8 kW Cu Kα ceramic X-ray tube and a PIXcel^3D^ 2 × 2 area detector, operating at 45 kV and 40 mA (after drop-casting onto silicon wafer). The XRD patterns were collected under ambient conditions using parallel beam geometry and symmetric reflection mode, and data analysis was conducted using the HighScore 4.1 software from PANanalytical.

Films with similar structure to device were characterized by Cary 5000 UV−vis−NIR spectrophotometer (Varian, Palo Alto, CA, USA), fluorescence spectrometer FLS920 (Edinburgh Instruments, Kirkton Campus, UK) at an excitation wavelength (Xenon lamp and monochromator) of 350 nm; A1 confocal fluorescence microscopy (Nikon, Shinjuku, Japan) with a laser excitation wavelength of 401 nm; and MFP3D AFM system with ORCA mode, with probes RMN-12PT400B (Bruker, Billerica, MA, US) coated with platinum. Different voltages of −3 V, −5 V and −7 V were applied on the tip. The sample used for C-AFM consisted of ITO/PEDOT:PSS/Poly-TPD/perovskite NCs, which follows similar structure to the device. In order to simulate the work mode of a full device, negative voltage was applied on the tip to inject electrons into the NCs while the ITO layer is electrically connected to the circuit to provide holes.

The cross-section of the device was measured by field-emission scanning electron microscope (SEM) JSM7500LA (Jeol, Tokyo, Japan) operating at 20 kV.

The current−voltage−luminance measurement was performed using a Keithley 2410 source-measure unit and an Agilent 34410A multi-meter coupled to a calibrated PDA 100A Si switchable gain detector from Thorlabs. The output of the Si detector was converted into power (photon flux) using a 50 Ω load resistance and the responsivity of the detector. The external quantum efficiency (EQE) was calculated as the ratio of the photon flux and the driving current of the device. The EL spectra of the devices were collected by an Ocean Optics HR4000+ spectrometer.

## 3. Results and Discussion

CsPbBr_3_ NCs were prepared with lead acetate trihydrate, cesium carbonate and benzoyl bromine as sources of lead, cesium and bromide, respectively. The synthesized CsPbBr_3_ NC solution was treated with different amount of SCN solution before purification with EA. According to the added amount of SCN solution including 0, 10, 30 and 50 µL, the CsPbBr_3_ NC solutions were labeled as 0SCN, 10SCN, 30SCN and 50SCN, respectively. The detailed treatment method can be found in the experimental section. Additionally, in the Supporting Information file, some routine characterization of the NCs is also included, such as TEM images (See Supplementary Figure S1a–j), showing an average size in the 10–12 nm range, and XRD patterns (Figure S1k), showing that all the XRD peaks are similar and can be assigned to CsPbBr_3_. The sulfur element contained in SCN was not detectable [37] by XPS and by HAADF-EDS in 30SCN. This may be due to either the sulfur contents being below detection threshold or to its probable overlap with the signal of lead.

**Figure 1:**
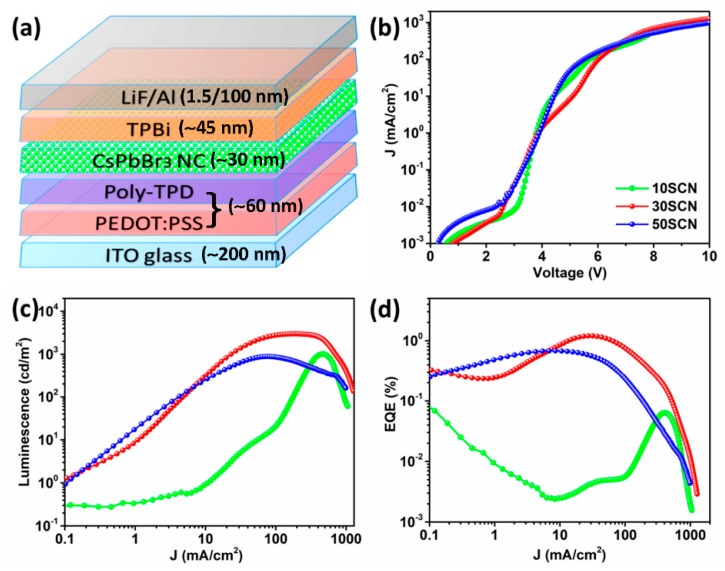
Characterization of LED performance. (**a**) Diagram of the LED structure; (**b**) current density as a function of driving voltage; (**c**) luminescence and (**d**) external quantum efficiency (EQE) as a function of current density in the NC LEDs.

With the treated CsPbBr_3_ NCs used as emissive material, we fabricated and characterized corresponding LEDs. Figure 1a presents a schematic diagram of the LED structure, ITO/PEDOT:PSS/poly-TPD/CsPbBr_3_ NC/TPBi/LiF/Al. The SEM cross-section of 30SCN device is shown in Figure S2. Figure 1b displays variation of current density with increasing voltage for the three devices. 50SCN presents higher leakage than 10SCN and 30SCN, suggesting higher conductivity of 50SCN NC film due to less organic than 10SCN and 30SCN NC films. At voltage higher than 7 V, 30SCN shows higher current density than 10SCN and 50SCN. In the current density plot of 30SCN, there is a turning point around 4.3 V, indicating variation of growth rate of the current density with increasing voltage (Figure S3a).

Figure 1c displays variation of luminance with increasing current density. It is clear that 30SCN shows higher luminance than 10SCN and 50SCN across the whole range of the current density and reaches the highest level of 2986 cd/m^2^ at 187 mA/cm^2^. 10SCN reaches the highest luminance of 1018 cd/m^2^ at 461 mA/cm^2^, and 50SCN reaches the highest luminance of 890 cd/m^2^ at 80 mA/cm^2^. 10SCN has the highest turn-on voltage at 4.4 V with luminance at 1cd/m^2^, while turn-on voltages of 30SCN and 50SCN are at 3.2 V and 3.3 V, indicating less insulating organic ligands in the CsPbBr_3_ NC film with increasing SCN additive during treatment. Although 30SCN shows a relatively slower decrease of luminance when the current density is less than 300 mA/cm^2^, all the three devices present luminance roll-off after reaching the respective highest luminance.

Figure 1d displays variation of EQE with increasing current density. 30SCN presents the highest EQE of 1.2% at 32 mA/cm^2^ or 5.5 V and the highest current efficiency of 4.42 cd/A among the three devices. The luminance at the maximum EQE is 1404 cd/m^2^, which is relatively high among the LEDs based on CsPbBr_3_ NCs with organic transporting layers (Table S1). In contrast, 10SCN and 50SCN show maximal EQEs of 0.06% at 380 mA/cm^2^ and 0.68% at 12 mA/cm^2^, respectively. Despite that, the trend of the EQE and current density is similar to the relation of luminance intensity and current density in Figure 1c; efficiency roll-off is more outstanding, especially in 30SCN and 50SCN. Given that current densities corresponding to the highest luminance or EQE of each device are different, it can be inferred that Auger recombination is not necessarily the reason for the efficiency roll-off in current cases. The device performance of 0SCN is displayed in Figure S4.

In order to explain the better device performance of the 30SCN, the NC solutions were spin-coated onto substrates coated with ITO/PEDOT:PSS/poly-TPD. The film samples show similar optical characterization results on the multi-layer substrates (see Figure S5).

**Figure 2:**
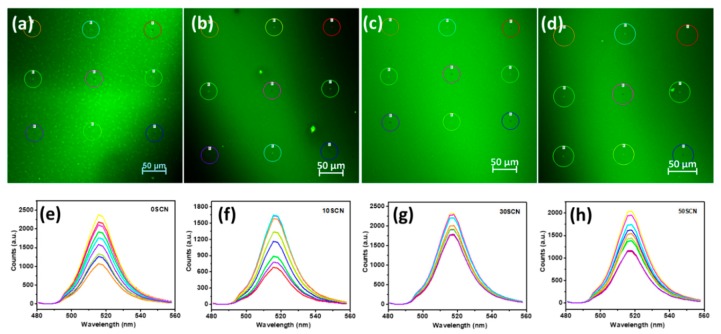
PL mapping images of NC films based on (**a**) 0SCN, (**b**) 10SCN, (**c**) 30SCN and (**d**) 50SCN NC solutions; (**e**–**h**) PL spectra of selected areas in each of the corresponding PL mapping images.

The film samples were then characterized by confocal microscopy. Figure 2 presents PL mapping images and PL spectra of selected areas on the images. The PL mapping image of 30SCN (Figure 2c) looks smoother and more homogeneous, in accord with the quantitative analysis result (Figure 2g) that the PL intensities of different selected areas with a diameter of 35 µm in 30SCN film sample show smaller difference or more homogeneous NC distribution than those of the other three film samples. In contrast, there are a few rather bright parts sparsely distributing in 10SCN and 50SCN film samples. The inhomogeneously bright areas may be caused by aggregation of NCs. Corresponding PL spectra of the selected areas in 10SCN and 50SCN film samples show relatively wide distribution in PL intensities, in accordance with the range of brightness showing in the PL mapping images. The spread in brightness can be regarded as striation defects and was demonstrated to be due to difference of surface tension and vapor pressure of organic residues and NCs coexisting in the film [19]. In other words, a proper ratio between ligands and CsPbBr_3_ NCs is beneficial for forming smooth NC film. This also indicates that the proportion of ligands and CsPbBr_3_ NCs in 30SCN is at an optimal level. In the PL mapping image of 0SCN (Figure 2a), one can clearly see bright dots distributing across the film, making the film look rough. This may be due to aggregation of the CsPbBr_3_ NCs or growth of some CsPbBr_3_ NCs, as demonstrated in another work in preparation showing that the untreated CsPbBr_3_ NCs in film are prone to grow into larger size due to desorption of ligands.

The above results on films match well with the LED performance. For example, too many ligands in 0SCN may explain the low current density and ineffective transport of injected carriers in the newly prepared LED, while there is strong leakage and emission from transporting layers, when large particles form after aging the device for some days (Figure S4). On the other hand, 10SCN shows the most inhomogeneous NC distribution on film, and the corresponding device displays the lowest efficiency. The 30SCN sample presents the best film homogeneity and the corresponding LED shows the highest luminance and EQE. Thus, the better device performance of the 30SCN may be attributed to both passivation effect of SCN, and the smooth and homogeneous NC film. Actually, both 30SCN and 50SCN gave good quality films, but 50SCN film was worse than 30SCN one. This may indicate that proper amount of SCN may have passivation effect and be beneficial, and too much SCN is not good. Thus, for 10 SCN and 30SCN, the difference in LED performance can be attributed to difference of film morphology or quality.

**Figure 3:**
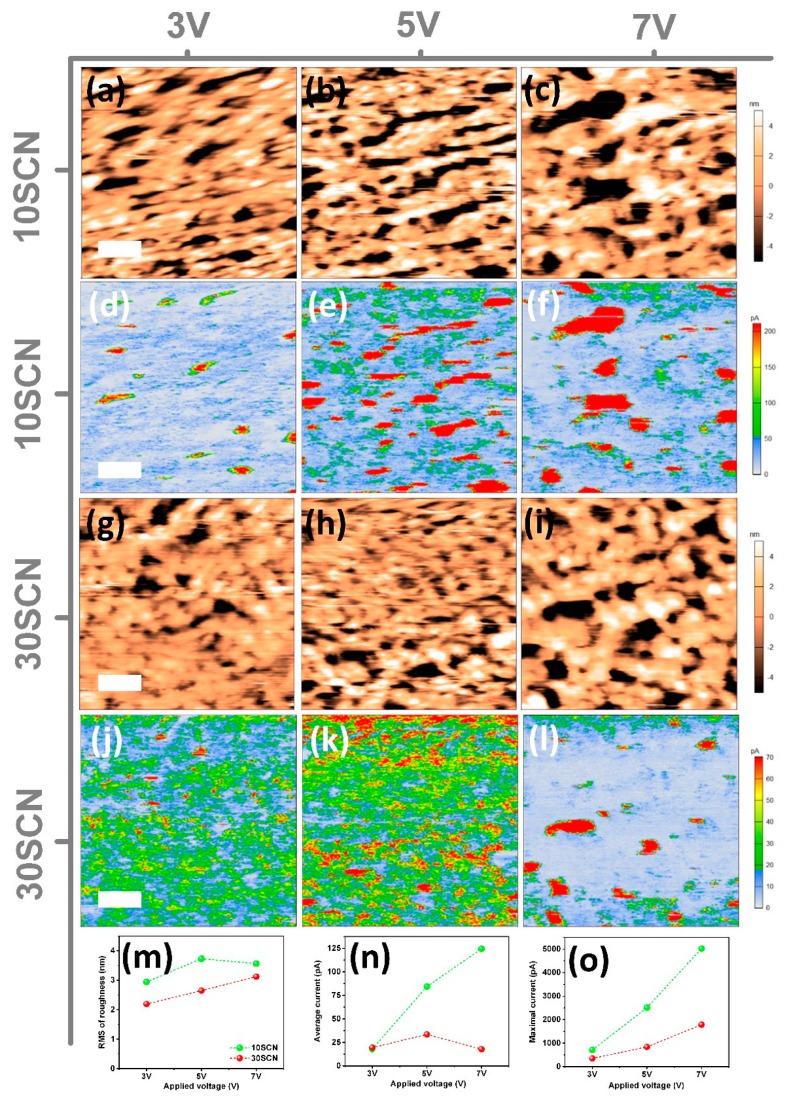
(**a**–**c**) Topography and (**d**–**f**) current mapping images of 10SCN NC film under 3 V, 5 V and 7 V; (**g**–**i**) topography and (**j**–**l**) current mapping images of 30SCN NC film under 3 V, 5 V and 7 V; comparison of (**m**) roughness, (**n**) average current, and (**o**) maximum current of 10SCN and 30SCN NC films under voltages. The scale bars are 200 nm.

Given that the above characterizations are performed on static films, we further tried to measure evolution of the films under voltage by conductive atomic force microscopy (C-AFM) [38]. Figure 3 shows topographical images and current mapping images of 10SCN and 30SCN under different applied voltages. In the case of 10SCN, with increasing applied voltages during scanning, the selected scanning area becomes rougher and aggregation of the CsPbBr_3_ NCs become increasingly serious. In specific, when the applied voltage increases from 3 V to 5 V, more areas without coverage of NCs come into being with the root mean square roughness parameter rising up from 2.9 to 3.7 nm (Figure 3a,b). When the voltage further increases to 7 V, the uncovered regions become larger in area in spite of the lesser number of domains (Figure 3c). The measured roughness apparently stays on the same level (~3.6 nm). Correspondingly, the current distribution follows the morphology evolution. At 3 V, the whole film is at a low current level and the average current is ~18 pA (Figure 3d). At 5 V, the overall current of the film increases according to the larger medium current (green yellow) and higher current (red) areas instead of lower current (blue white) areas (Figure 3e). The red areas of the current mapping image indicate higher current leakage than the other parts and may represent absence of CsPbBr_3_ NCs, in line with the morphology image. When 7 V is applied during scanning, the distribution of red and blue-white domains change (Figure 3f). The red domains decrease in number and become larger in size, while the rest of the scanning area mainly lies on the blue-white current levels. This suggests stronger leakage through the uncovered areas and less current flow through the NC areas, supplying evidence for low efficient radiative recombination of injected carriers. This may explain the low luminance and EQE of 10SCN.

In the case of 30SCN, the overall roughness of the film is less than that of the 10SCN at different voltages (Figure 3g–i). With applied voltages increasing from 3 V to 7 V, the roughness of the 30SCN film increases monotonously from 2.2 nm to 3.1 nm (Figure 3m). A few large regions without coverage of NCs appear (Figure 3l), especially at 7 V. The corresponding current images show a rather different appearance from that of 10SCN. At 3 V, the current image is mainly occupied by blue-white and green-yellow regions (Figure 3j). At 5 V, small red domains start to increase and green-yellow spots dominate the image (Figure 3k), suggesting efficient carrier injection into NCs. This is in accord with the optimal device performance near 5 V. At 7 V, most area of the image is blue-white with a few large red spots (Figure 3l), suggesting enhanced leakage at high voltage and lower radiative recombination efficiency at high current density. This is in line with the decreased average current from ~33 pA to ~18 pA (Figure 3n), while the maximum current increases from ~0.8 nA to ~1.8 nA when the voltage is increased from 5 V to 7 V (Figure 3o). The formation of large uncovered regions together with localized high current density at 7 V may explain the efficiency roll-off at voltage higher than 6 V in 30SCN LED. Thus, morphology deterioration of NC film with increasing applied voltage, which may lead to enhanced leakage, could be a reason for efficiency roll-off in LED at high current density [39], as predicted in a report [35] on CsPbBr_3_ thin film-based LED. Furthermore, stable morphology of NC film or stable NC distribution in NC film is expected to be able to relieve efficiency roll-off. This is in agreement with some reports [13,40] on embedding perovskite in a matrix, in which the perovskite NCs or particles were fixed in the matrix and morphologies of the films are more stable during operation.

Comparing the average current of 10SCN and 30SCN samples at different voltages, it is clear that the initial average currents at 3 V are almost the same (Figure S6). However, when the current increases to 5 V, the average current in 10SCN significantly rises from ~18 pA to ~84 pA with a large uncertainty range (Figure S6), while the average current in 30SCN increases only from ~19 pA to ~33 pA (Figure 3n). With higher voltage of 7 V, the average current further increases to ~125 pA in 10SCN and decreases to ~18 pA in 30SCN. The maximum current follows a similar trend to the average current (Figure 3o). In respect of 10SCN sample, the maximum current reaches ~2.5 nA and ~5 nA at 5 V and 7 V, respectively. In contrast, the maximum current is merely ~1.8 nA at 7 V in the 30SCN sample. Thus, the higher average current together with uncovered regions in 10SCN may be the reason for the lower device performance of 10SCN than that of 30SCN, in which the current increases more smoothly and few uncovered large regions are formed when the applied voltage is less than 7 V.

**Figure 4:**
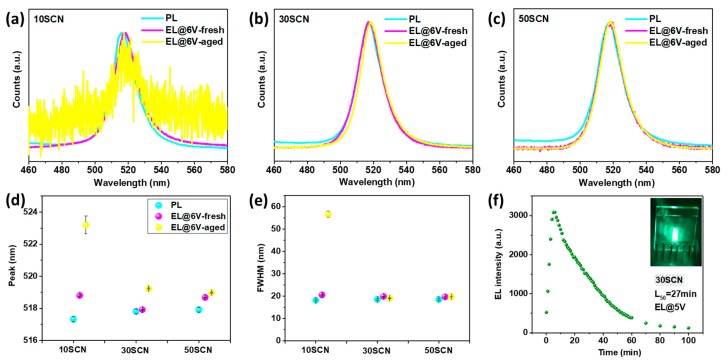
Comparison of PL and EL spectra of (**a**) 10SCN, (**b**) 30SCN, and (**c**) 50SCN NC films and fresh as well as aged devices; Comparison of (**d**) PL and EL peaks; and (**e**) FWHM of the PL and EL peaks; (**f**) Evolution of EL intensity as a function of running time of the 30SCN aged device. The inset is a capture of the device during the operative stability test.

The stability of the devices was also studied. Figure 4 presents EL under applied voltage of 6 V from both fresh and aged devices of 10SCN, 30SCN and 50SCN. With PL spectra of fresh films as comparison, it appears that the EL peak of the fresh 30SCN device is the same as corresponding PL peak (Figure 4b). The EL peaks of the fresh 10SCN and 50SCN devices are 1.5 nm and 0.8 nm red-shifted with respect to their PL peaks (Figure 4a,c), respectively. When comparing the EL peaks of both fresh and aged devices, all devices display a stable EL peak position or exhibit a red shift (Figure 4d). For instance, for the 10SCN devices, there is a red shift from ~519 nm to ~523 nm on aging. On the other hand, the 30SCN devices show only a minor red-shift, if any, from ~518 nm to ~519, and the 50SCN devices exhibit no red shift at all, suggesting that more SCN within the considered range is favorable for stability of CsPbBr_3_ NCs in the device. In addition, the FWHMs of the EL peaks of 30SCN and 50SCN show negligible change between the fresh and aged devices (Figure 4e). The stable EL spectra of the 30SCN and 50SCN devices at 6 V are also reflected in the CIE coordinate system (Figure S7), indicating that the treated NCs in the 30SCN and 50SCN devices are rather stable even after storage for four months.

Furthermore, EL peaks and corresponding FWHM at different applied voltages were analyzed for the fresh and aged devices (Figure S8a,b). There are slight blue shifts in the EL peaks and gradual increases in the FWHM with increasing applied voltages in the fresh devices, which could be attributed to a field-induced quantum confinement Stark effect and an enhanced longitudinal optical-phonon coupling at high external field [5,41], respectively. In the case of the aged devices, the FWHM of the EL peaks in 10SCN and 30SCN devices decrease slightly with increasing voltage, while it increases slightly in 50SCN. The EL spectra of the fresh and aged 30SCN devices at different voltages are shown in Figure S8c,d.

The performance of the aged devices were characterized and the statistic results are shown in Figure S9. The aged 30SCN devices still show a higher average EQE than those of 10SCN and 50SCN aged devices. In particular, the champion EQE of the aged 30SCN is 1.9% at 5.6 V, higher than that of the fresh champion device (Figure S4). The higher EQE of the aged device is due to lower current density. According to our research on conductivity of fresh and aged NC films, the aged NC films show a relatively higher conductivity than the fresh devices, which suggests assignment of the decreased current density in the aged devices to degradation of organic transporting layers and corresponding decreased carrier mobility. Statistic results on performance of 41 aged devices based on 30SCN CsPbBr_3_ NCs but with variation in spin coating speed of different layers are shown in Table S2. These results confirm the high stability of the 30SCN devices.

Additionally, operational stability of the 30SCN aged device was measured at 5 V. There is an initial increase in the EL intensity within the first 6 min (Figure 4f), then the EL intensity shows an exponential deterioration. The half-lifetime (L50) is ~27 min, showing competitive stability in CsPbBr_3_ NC-based LED (Table S3). EL peaks collected every one minute (Figure S10) are shown to stably fluctuate around 518 nm throughout the measurement, indicating stable emission property of the 30SCN aged device. Inset of the Figure 4f displays the running device during the measurement.

## 4. Conclusions

In summary, green LEDs were fabricated based on CsPbBr_3_ NCs synthesized with lead acetate and benzoyl bromide as source of lead and bromine. Softer Lewis acid, ammonium thiocyanate, was used to treat CsPbBr_3_ NCs before purification with polar solvent. The 30SCN device showed the best performance, and we investigated possible reasons for the score of this champion device. Morphology and current distribution mapping of NC film under applied voltages were studied by C-AFM. The results show that performance of NC-based LEDs strongly depends on the distribution of NCs in the emissive layer, and that the efficiency roll-off in the NC-based LEDs at high current density can be attributed to displacement and deterioration of NC film with increasing applied voltages. Characterization of fresh and aged devices as well as measurement of running time at 5 V shows relatively high stability of the 30SCN device. The current work proposes a facile route to treat sensitive perovskite NCs and to fabricate perovskite NC-based LED with high stability. Moreover, a new evidence was supplied for the efficiency roll-off in NC LED.

## Supplementary Materials

The following are available online at https://www.mdpi.com/1996-1944/13/2/367/s1. Figure S1. TEM images and size distributions of NCs in (a,b) 0SCN, (c,d) 10SCN, (e,f) 30SCN, and (g–k) 50SCN NC solutions. Comparison of (i) fitted average size with FWHM of fitting peaks as error. (j) XRD patterns of the NCs; Figure S2. SEM cross-section of (a) 10SCN; (b) 30SCN; and (c) 50SCN devices. From bottom to top, the layers include ITO /PEDOT:PSS + poly-TPD (~60 nm) /CPB NC (~30 nm) /TPBi (~45 nm); Figure S3. (a) Current density of 30SCN device as a function of driving voltage; (b) EL spectra of 30SCN device at different driving voltages; Figure S4. Comparison of (a) current density as a function of driving voltage; (b) EQE, (c) luminescence of 0SCN and 30SCN fresh and aged devices as a function of current density; (d) EL spectra of 0SCN and 30SCN aged devices; Figure S5. (a) Visible light absorption plots; (b) PL; (c) decay lifetime; and (d) fitted decay lifetime and PLQY of NC films on PEDOT:PSS/Poly-TPD substrates; Figure S6. Comparison of average current of 10SCN and 30SCN NC films at different applied voltages; Figure S7. EL positions of fresh and aged devices in a CIE coordinate system; Figure S8. Comparison of (a) EL peaks and (b) FWHM of the EL peaks of fresh and aged devices. EL spectra of 30SCN (c) fresh and (d) aged devices; Figure S9. Statistics of (a) maximum EQE, (b) maximum luminescence, (c) maximum current efficiency, and (d) current density at the maximum EQE of aged devices; Figure S10. (a) EL spectra and (b) evolution of the EL peaks collected during operation stability test of 30SCN aged device; Table S1. Summary of LED performance in literature; Table S2. Statistics of LED performance of the fresh and aged devices; Table S3. Statistics of performance of 41 aged 30SCN-based LEDs.
