# Supplementary material for: Thiocyanate-Treated Perovskite-Nanocrystal-Based Light-Emitting Diodes with Insight in Efficiency Roll-Off
Source: Materials (Basel). 2020 Jan 13;13(2):367. doi: 10.3390/ma13020367 (PMC7014121; doi:10.3390/ma13020367)
Supplement: Supplementary file 1 [file materials-13-00367-s001.pdf]

Supplementary Materials

Thiocyanate-Treated Perovskite-Nanocrystal-Based Light-Emitting Diodes with Insight in Efficiency Roll-Off

Fang Chen [1,2], Karunakara Moorthy Boopathi [3], Muhammad Imran [3], Simone Lauciello [3] and Marco Salerno [4,*]

1 Optoelectronics, Istituto Italiano di Tecnologia, via Morego 30, 16163 Genova, Italy; fang.chen@iit.it
2 Dipartimento di Chimica e Chimica Industriale, Università degli Studi di Genova, via Dodecaneso 31, 16146 Genova, Italy
3 Nanochemistry Department, Istituto Italiano di Tecnologia, via Morego 30, 16163 Genova, Italy; karunakara.boopathi@iit.it (K.M.B.); muhammad.imran@iit.it (M.I.); simone.lauciello@iit.it (S.L.)
4 Materials Characterization Facility, Istituto Italiano di Tecnologia, via Morego 30, 16163 Genova, Italy
* Correspondence: marco.salerno@iit.it; Tel.: +39-010-2896-885

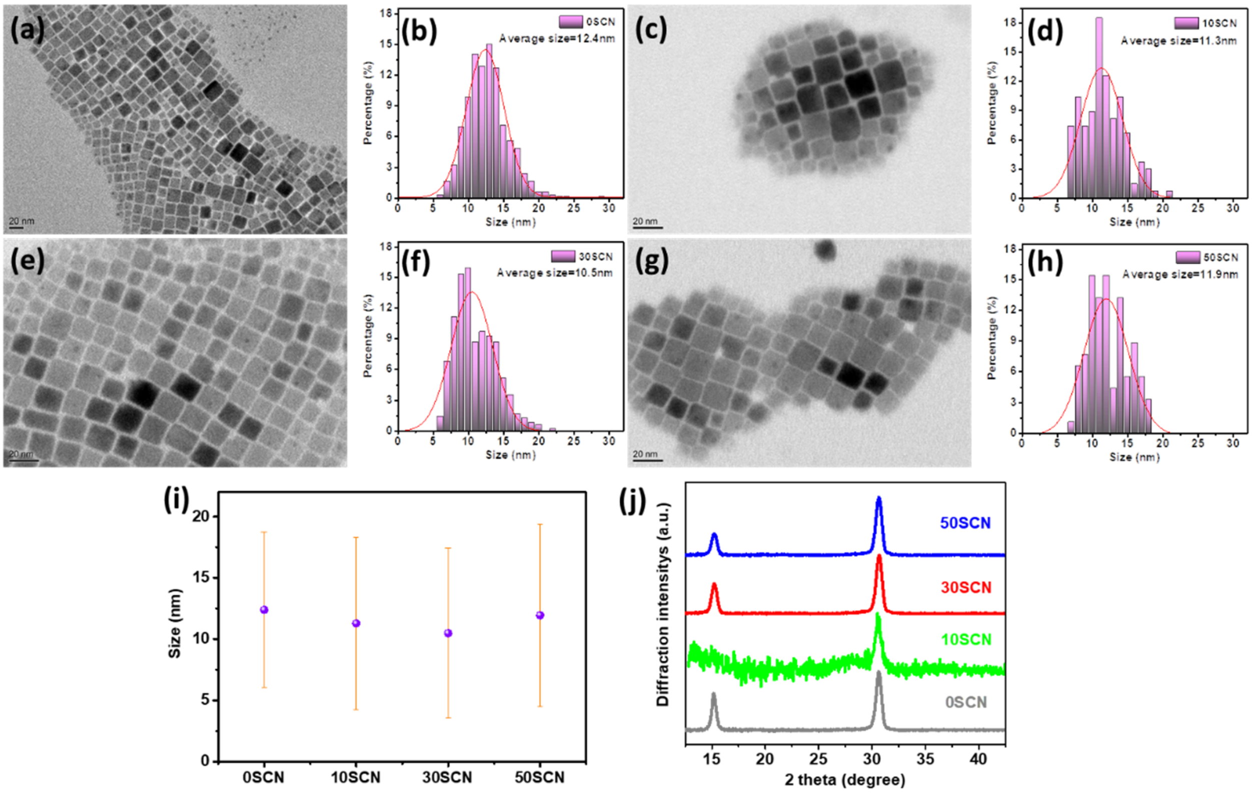

Figure S1. TEM images and size distributions of NCs in (**a**,**b**) 0SCN, (**c**,**d**) 10SCN, (**e**,**f**) 30SCN, and (**g**–**k**) 50SCN NC solutions. Comparison of (**i**) fitted average size with FWHM of fitting peaks as error. (**j**) XRD patterns of the NCs.

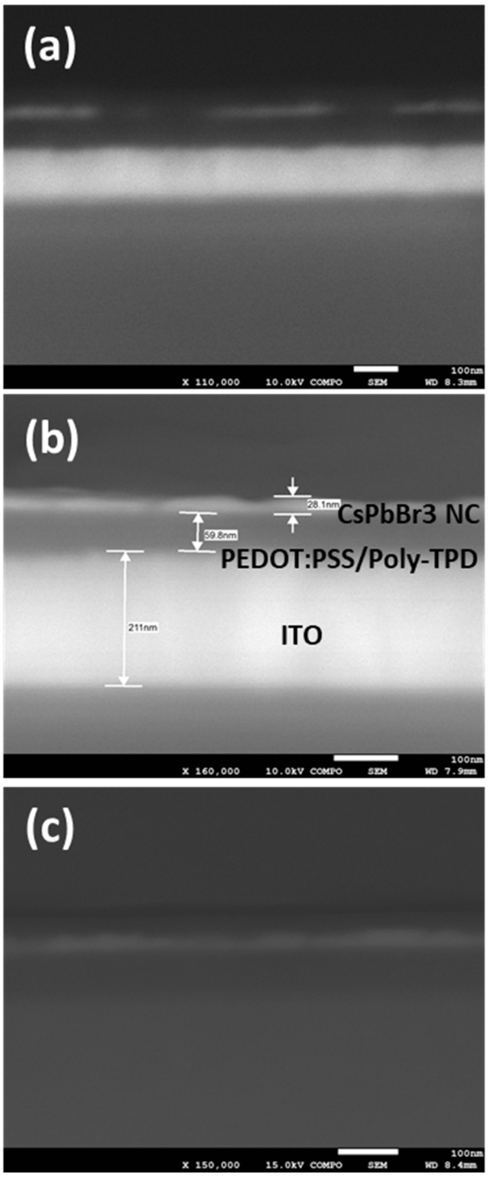

Figure S2. SEM cross-section of (**a**) 10SCN; (**b**) 30SCN; and (**c**) 50SCN devices. From bottom to top, the layers include ITO /PEDOT:PSS + poly-TPD (~60 nm) /CPB NC (~30 nm) /TPBi (~45nm).

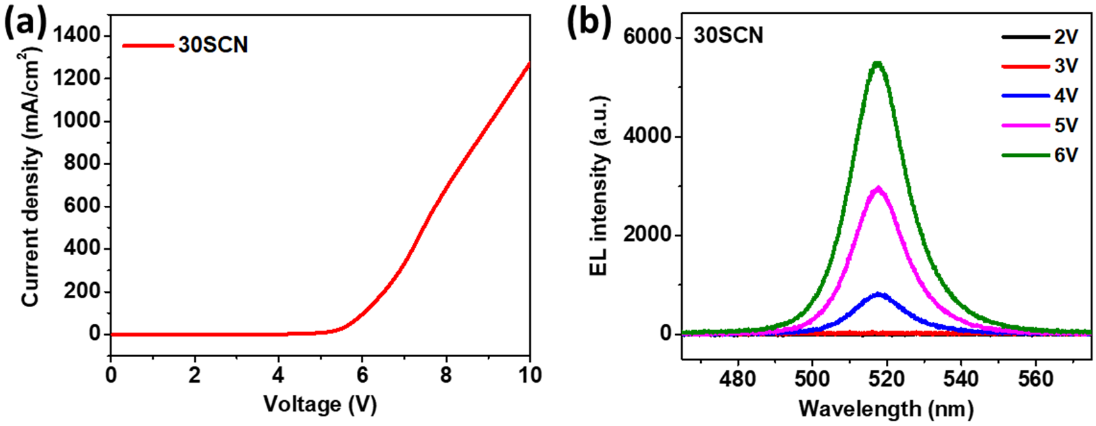

Figure S3. (**a**) Current density of 30SCN device as a function of driving voltage; (**b**) EL spectra of 30SCN device at different driving voltages.

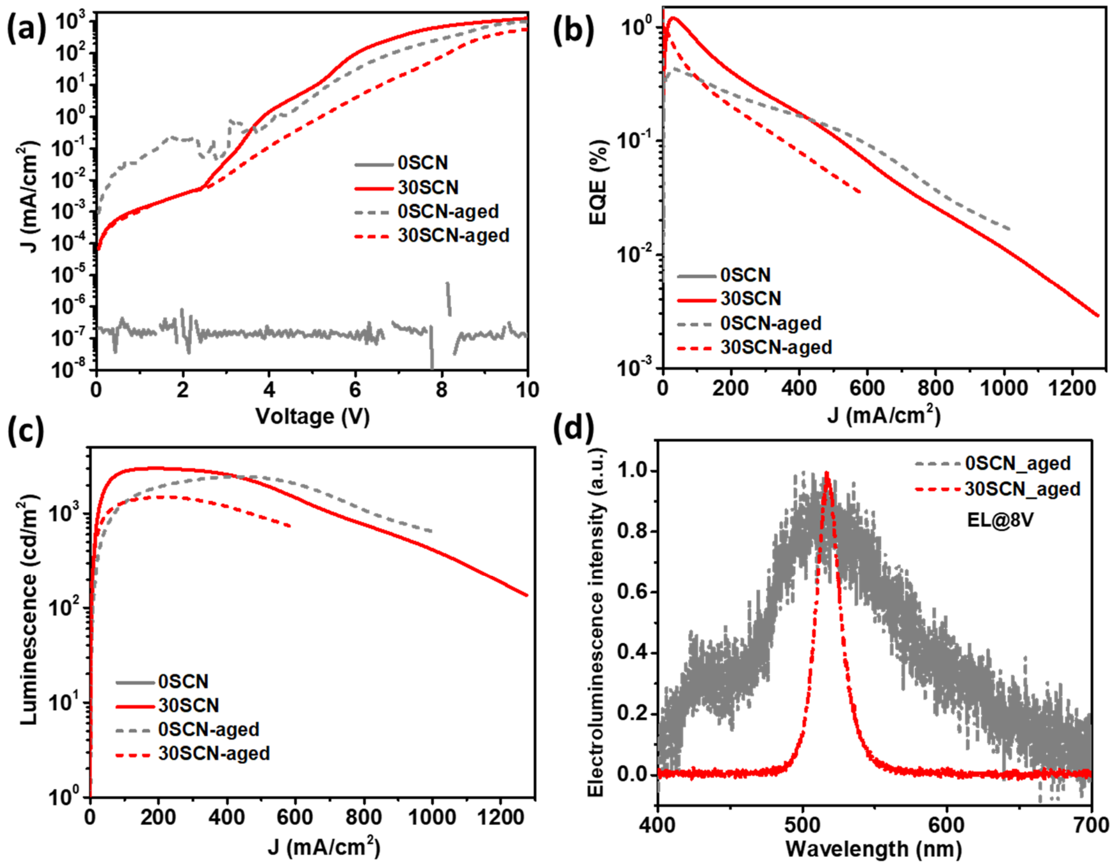

Figure S4. Comparison of (**a**) current density as a function of driving voltage; (**b**) EQE, (**c**) luminescence of 0SCN and 30SCN fresh and aged devices as a function of current density; (**d**) EL spectra of 0SCN and 30SCN aged devices.

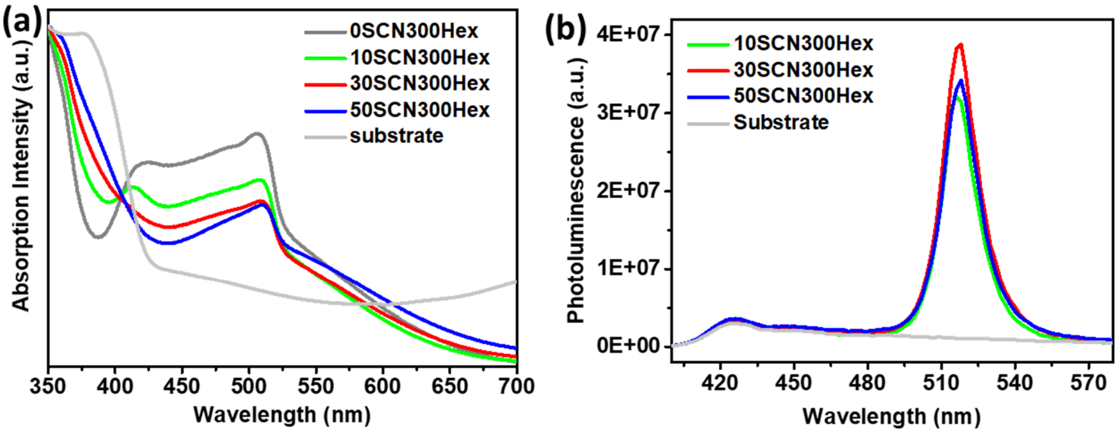

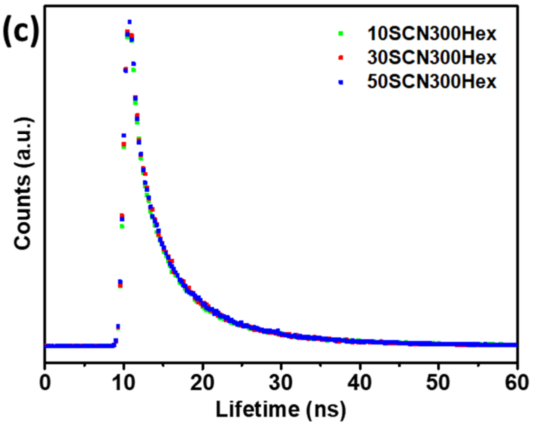

(d)

	10SCN300 Hex	30SCN300 Hex	50SCN300 Hex
t1	1.66875	1.68963	1.69124
t1%	76.6%	74.0%	75.7%
t2	7.79257	7.626	7.87683
t2%	23.4%	26.0%	24.3%
tave	5.27	5.33	5.39
PLQY	18.29%	13.94%	14.63%

Figure S5. (**a**) Visible light absorption plots; (**b**) PL; (**c**) decay lifetime; and (**d**) fitted decay lifetime and PLQY of NC films on PEDOT:PSS/Poly-TPD substrates.

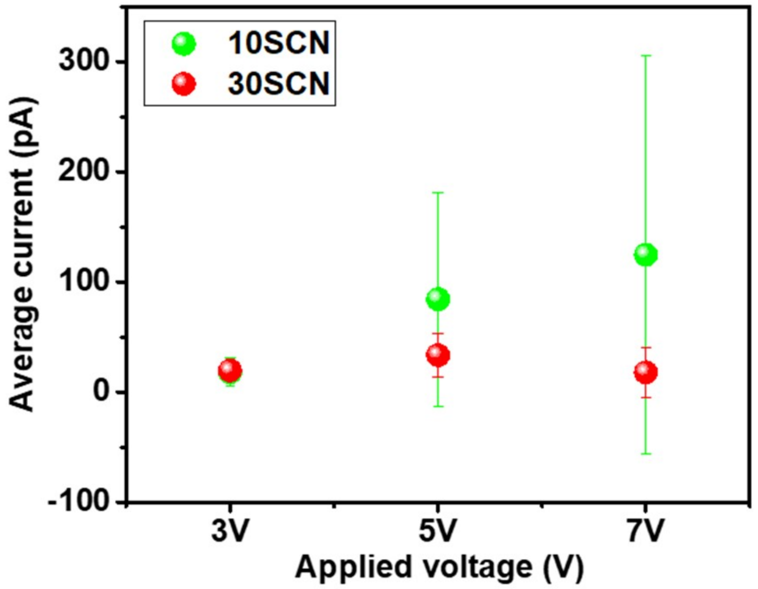

Figure S6. Comparison of average current of 10SCN and 30SCN NC films at different applied voltages.

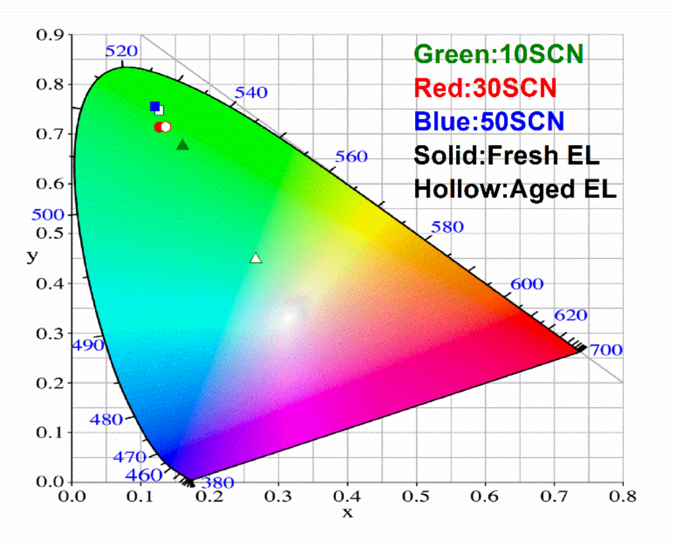

Figure S7. EL positions of fresh and aged devices in a CIE coordinate system.

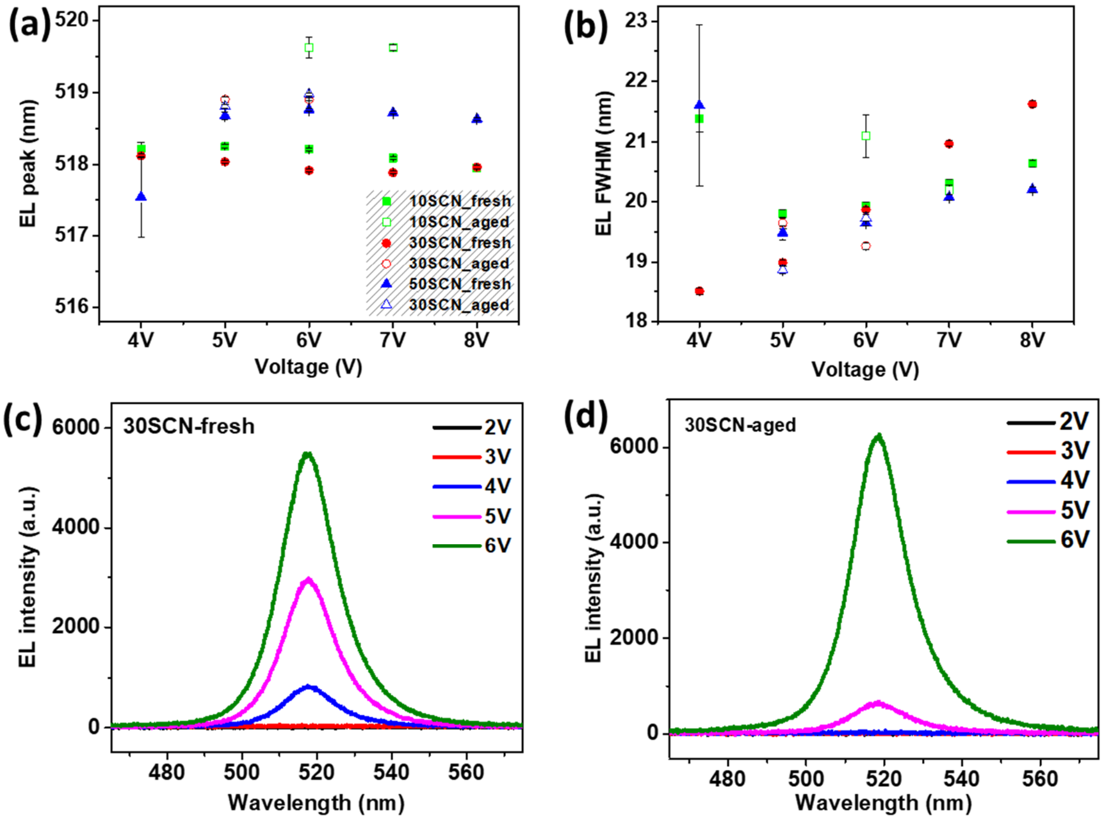

Figure S8. Comparison of (**a**) EL peaks and (**b**) FWHM of the EL peaks of fresh and aged devices. EL spectra of 30SCN (**c**) fresh and (**d**) aged devices.

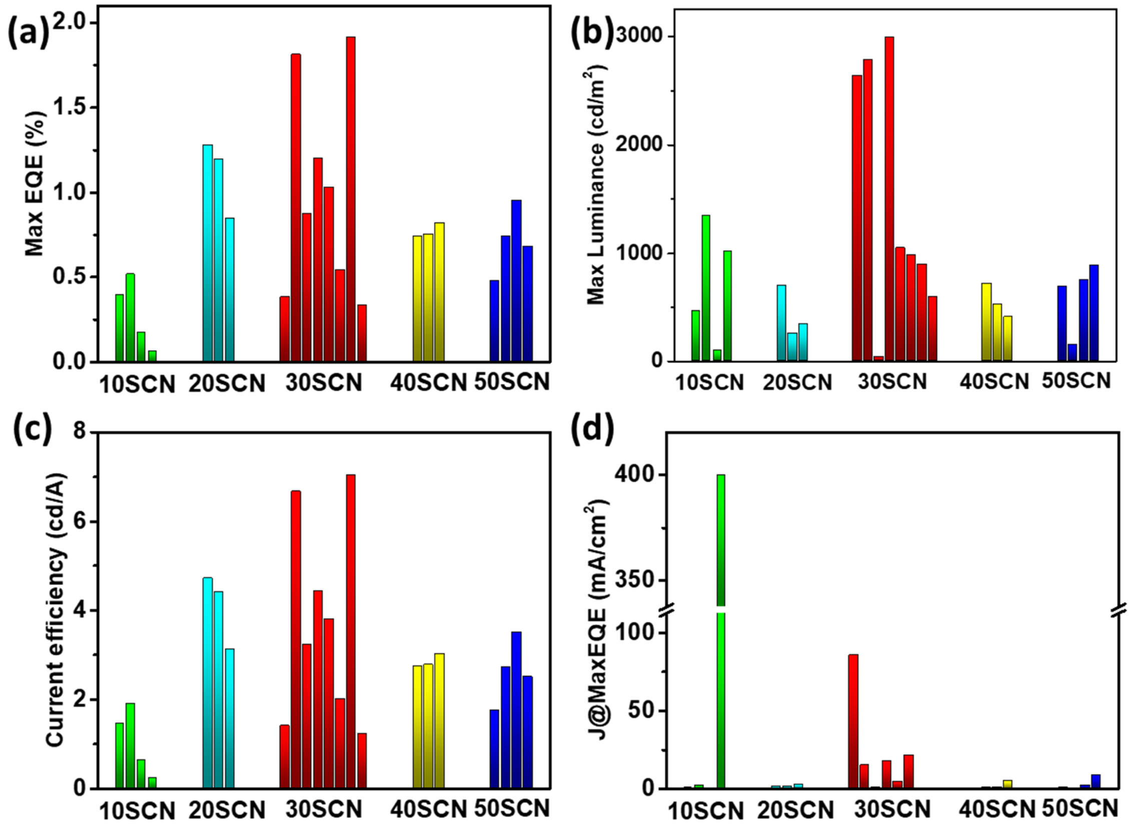

Figure S9. Statistics of (**a**) maximum EQE, (**b**) maximum luminescence, (**c**) maximum current efficiency, and (**d**) current density at the maximum EQE of aged devices.

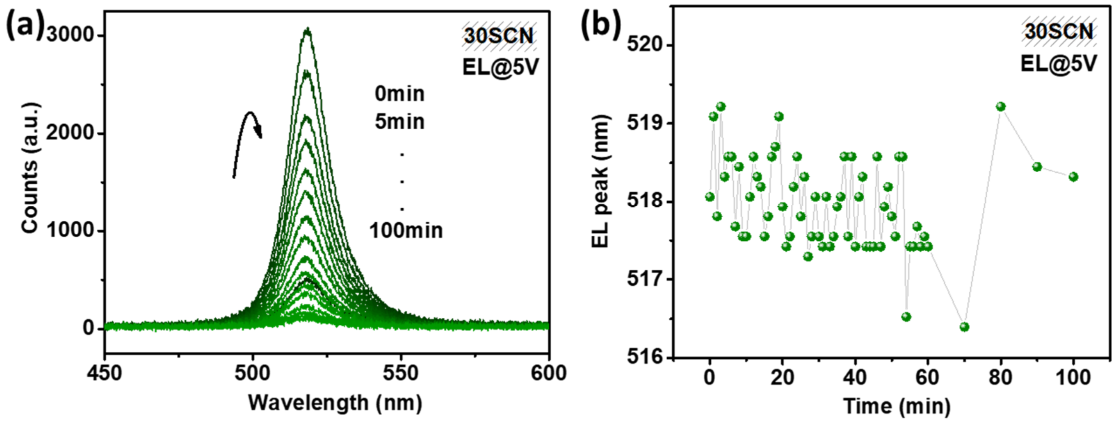

Figure S10. (**a**) EL spectra and (**b**) evolution of the EL peaks collected during operation stability test of 30SCN aged device.

Table S1 Summary of LED performance in literature.

Year	Material	L50	Max EQE(%)	Lum@Max EQE (cd/m2)	Current density@ Max EQE (mA/cm2)	Voltage @Max EQE (V)	Turn-on voltage @ 1cd/m2	Max Current efficiency (cd/A)	Max Luminance (cd/m2)	EL peak	EL FWHM	Source
	CsPbBr3 NCs	27min@5V	1.2	1404	31.8	5.50	3.2	4.42	2986	518	19	This work
2015	CsPbBr3 QD		0.12	~100	~140	~7	6.5	0.43	946	516	23	Adv. Mater. 2015, 27, 7162–7167
2016	CsPbBr3 QD		0.33	~100	~20	~5	3.8		934	510	25	Advanced Functional Materials, 2016. 26(47): p. 8757-8763.
2016	CsPbBr3 QD		0.19	~200	~30	~4.3	2.6		2335	523	19	Advanced Materials, 2016. 28(18): p. 3528-3534.
2016	DDAB-OA-CsPbBr3 QDs		3	~10	~0.05	4.50	3	<10	330	515	19	Advanced Materials, 2016. 28(39): p. 8718-8725.
2016	CsPbBr3 QD	10min@5V	0.06	~100	~100	>5	3.5	0.19	1377	516	19	Nano Lett. 2016, 16, 1415–1420
2016	CsPbBr3-CsPb2Br5 Composites		2.21	~2200	~5	8.6	4.6	8.98	3853	527	24	Advanced Functional Materials, 2016. 26(25): p. 4595-4600.
2017	DDAB-capped CsPbBr3 QD		8.73	<30	~0.01	~2.8	2.6	~20	1660	512	17	ACS Appl. Mater. Interfaces 2017, 9, 18054–18060
2017	CsPbBr3 QD		6.27	~3000	<100	~8	3.4	13.3	15185	512	20	Advanced Materials, 2017. 29(5): p. 1603885.
2017	CsPbBr3 QD		1.19	~2000	<100	~6	4.6	3.1	12090	515	18	Chemistry of Materials, 2017. 29(12): p. 5168-5173.
2017	CsPbBr3 nanoplate		1.1	~100	~1	<5	3.5		590	520	17	ACS Nano, 2017. 11(10): p. 10206-10213.
2017	CsPbBr3 thin film		5.34	~300	~900	~5.2	2.5	19	36600	522	16	The Journal of Physical Chemistry Letters, 2017. 8(17): p. 4148-4154.
2017	CsPbBr3 QD	>600min	2.39					2.25	3809	522		Journal of Materials Chemistry C, 2017. 5(18): p. 4565-4570.
2018	DDAB-capped CsPbBr3 QD	27min@3.5V	0.58	~355	~15	4.5	2.5	0.62	355	517		Adv. Optical Mater. 2018, 1800007
2018	DDAB-capped CsPbBr3 QD		8.08	1	~0.01	~3	3	25.1	<200	510	20	ACS Applied Materials & Interfaces, 2018
2018	Zwitterionic-capped CsPbBr3 NCs		2.5	1641	21.7	3.5	~2.5	7.5	24000	516	16	ACS Energy Letters, 2018. 3(3): p. 641-646.
2018	4-layered CsPbBr3 NCs		1.3	~6	~0.2	6.50	~4.5	5.2	54	~520	20	The Journal of Physical Chemistry C, 2018. 122(25): p. 13767-13773.
2018	PEABr-capped CsPbBr3 NCs		4.33	~4000	~1000	4.50	2.66	13.43	12650	514		Nano Research, 2018.
2018	CsPbBr3 NCs			~25				3.72	106	~518		Nanoscale, 2018. 10(18): p. 8591-8599.
2018	CsPbBr3 NCs		3.79	5863.5	~90	9.00	~2.8	7.96	6093.2	519	19	ACS Nano, 2018. 12(2): p. 1462-1472.
2018	CsPbBr3 NCs	>3600min@8V	4.63	~10000	~350	~10		8.736	10206	518.5		Advanced Functional Materials, 2018. 28(20): p. 1707031.
2018	TOAB/DDAB/OTAc-capped CsxFA1-xPbBr3		11.6	~400	~1	~3.2	2.75	45.4	55800	515	18	Advanced Materials, 2018. 30(30): p. 1800764.
2018	DDAB-capped ZnBr2-treated CsxFA1-xPbBr3 NCs	136min@~2.8V	16.48	~400	~0.6		2.4	66.7	76940	518	18	Adv Mater, 2018. 30(50): p. e1805409.
2018	OPA-capped CsPbBr3 NCs	30min@~4V	6.5	~400	~2	~3.9	2.8	18.13	7085	516	19	ACS Applied Materials & Interfaces, 2018. 10(4): p. 3784-3792.
2018	crown ethers-shelled CsPbBr3 NCs (single-layer)		1.27	~3500	~25	~4.2	2.5	4.29	14454	519		ACS Energy Letters, 2018. 3(3): p. 526-531.
2018	crown ethers-shelled CsPbBr3 NCs (double-layer)	9.25min@~5V	2.64	~250	~1	~4	2.5	9.22	3880	519		ACS Energy Letters, 2018. 3(3): p. 526-531.
2018	CsPbBr3 NCs	10.5min@10V	1.7	~1000	<0.1	>10		5.57	1562	522	16	ACS Applied Materials & Interfaces, 2018. 10(23): p. 19828-19835.
2018	CsPbBr3 NCs		5.7	~12000	<1000	~6	2.3	19.9	46000	517	<20	Frontiers in Chemistry, 2018. 6(381).
2018	CsPbBr3–CsPb2Br5 NCs	120min@5.5V	~0.6	~5000	~100	~5.2	2.5		8383	518	19	Nanoscale, 2018. 10(41): p. 19262-19271.
2019	CsPbBr3 | Cs4PbBr6 thin film	>420min	2.25		~250		2.9	~7.2	53486	519	23	Advanced Functional Materials, 2019. 29(5): p. 1807345.
2019	CsPbBr3-Cs4PbBr6 NCs		1.21	~100		8.10			1941.6	515	16.3	Journal of Materials Chemistry C, 2019. 7(25): p. 7548-7553.
2019	CsPbBr3 thin film	15000min@~3.8V	10.5	~10000	~20	~4.5	2.8	32	16436	518	19	Nature Communications, 2019. 10(1): p. 665.

Table S2. Statistics of LED performance of the fresh and aged devices.

Sample	Type	EQE (%)	Current Efficiency (cd/A)	Luminance (cd/m²)
10SCN	fresh	0.06	0.23	1018.4
	aged	0.52	1.91	1347.3
30SCN	fresh	1.20	4.42	2986.3
	aged	1.91	7.04	898.9
50SCN	fresh	0.68	2.51	890.4
	aged	0.95	3.51	747.6

Table S3. Statistics of performance of 41 aged 30SCN-based LEDs.

-	Max EQE (%)	Voltage@Max EQE (V)	Current Efficiency (cd/A)	Luminance (cd/m²)	J@Max EQE (mA/cm²)
Average	0.7	5.7	2.6	696.5	22.0
Maximum	1.9	5.6	7.0	898.9	4.4

© 2020 by the authors. Licensee MDPI, Basel, Switzerland. This article is an open access article distributed under the terms and conditions of the Creative Commons Attribution (CC BY) license (http://creativecommons.org/licenses/by/4.0/).
